# Renal Safety of a Tenofovir-Containing First Line Regimen: Experience from an Antiretroviral Cohort in Rural Lesotho

**DOI:** 10.1371/journal.pone.0017609

**Published:** 2011-03-02

**Authors:** Helen Bygrave, Katharina Kranzer, Katherine Hilderbrand, Guillaume Jouquet, Eric Goemaere, Nathalie Vlahakis, Laura Triviño, Lipontso Makakole, Nathan Ford

**Affiliations:** 1 Médecins Sans Frontières, Morija, Lesotho; 2 London School of Hygiene and Tropical Medicine, London, United Kingdom; 3 Médecins Sans Frontières, Cape Town, South Africa; 4 Centre for Infectious Disease Epidemiology and Research, University of Cape Town, Rondebosch, South Africa; 5 Scott Hospital, Morija, Lesotho; University of Cape Town, South Africa

## Abstract

**Introduction:**

Current guidelines contraindicate TDF use when creatinine clearance (CrCl) falls below 50 ml/min. We report prevalence of abnormal renal function at baseline and factors associated with abnormal renal function from a community cohort in Lesotho.

**Methods:**

We calculated changes in CrCl from baseline for patients initiated on TDF at 6 and 12 months and the proportion of patients initiated on TDF who developed renal impairment. Screening algorithms were developed using risk factors determined by multivariate analysis.

**Results:**

Among 933 adults for whom baseline creatinine was available, 176 (18.9%) presented with a baseline CrCl <50 ml/min. Renal function improved during follow-up. 19 patients who developed renal toxicity during follow up remained on TDF; renal function improved (CrCl≥50 ml/min) in all but 3 of these patients. Among 15 patients with a baseline CrCl <50 ml/min were started in error, none developed severe renal impairment.

**Conclusion:**

In this setting TDF-associated renal toxicity is rare and mainly transient. Further studies to assess TDF safety at lower CrCl thresholds are warranted.

## Introduction

In 2009 the World Health Organisation revised its guidelines for antiretroviral therapy (ART) in resource-limited settings to recommend replacing stavudine with either tenofovir or zidovudine [1]. In randomized trials tenofovir has demonstrated comparable efficacy when compared with other first-line regimens containing stavudine, zidovudine or abacavir [2]–[4], and has the additional advantage of low toxicity and availability as a once-daily, fixed-dose generic formulation [5]. However, concerns about potential nephrotoxicity have led to recommendations for close clinical monitoring. Clinical detection of renal impairment is difficult in resource-limited settings. Early stages of renal dysfunction are assessed through laboratory monitoring of creatinine and glomerular filtration rate is calculated using either the Cockcroft Gault formula or the modification of diet in renal disease (MDRD) calculation.

Current international guidelines contraindicate TDF use when creatinine clearance (CrCl) falls below 50 ml/min unless dose reductions are made [6] and acknowledging the potential for nephrotoxicity, six-monthly monitoring of renal function is recommended by national guidelines [7]. If TDF is to be implemented in resource-limited settings where numbers requiring antiretroviral therapy are high but access to laboratory services are scarce, these monitoring protocols may need to be further simplified.

In this paper we report on program performance, early toxicity outcomes, prevalence of abnormal renal function at baseline, and factors associated with abnormal renal function from a community cohort in Lesotho.

## Methods

### Study setting

Médecins Sans Frontières (MSF) and the Ministry of Health and Social Welfare established a decentralized HIV/AIDS care and treatment programme at the primary health care level in Scott Health Service Area, Lesotho in 2006. ART services are provided across 14 primary care health centres and one district hospital, each staffed by nurses who initiate and manage ART, with complicated cases referred to the district hospital [8]. National guidelines recommend prescribing TDF to all non-pregnant adults above 18 years of age with adequate renal function, defined as a CrCl >50 ml/min calculated using the Cockcroft-Gault formula.

### Data collection

All adults initiating antiretroviral therapy from 1 January 2008 to 31 December 2008 and who had a baseline serum creatinine were included in the analysis. Data were extracted from clinic registers and medical notes by a team of three clinicians, entered into an Access database then exported into STATA (version 11) for analysis.

### Creatinine clearance and renal function

Creatinine measurements were performed in the district hospital laboratory. Crcl was calculated using the Cockcroft Gault formula using weight taken on the same day that creatinine measurements were done but without adjusting for body surface area, consistent with guidelines for resource-limited settings. Baseline renal function was categorized according to CrCl as follows: normal (CrCl ≥90 ml/min), mild (CrCl 60–89 ml/min), moderate (CrCl 30–59 ml/min) and severe renal impairment (CrCl <30 ml/min). Baseline renal function was further categorized into CrCl <50 ml/min and ≥50 ml/min. CrCl results that had been calculated and documented by the nurses were extracted from the clinical records.

### Data analysis

The proportion of creatinine measurements performed according to the monitoring protocol was calculated along with the proportion of CrCl documented in the notes. Agreement between nurse- and computer-calculated CrCl was determined by stratifying the nurse-calculated CrCl as less or greater than 10% different from the computer-calculated CrCl. Improvement over time was assessed by the χ^2^ test.

Baseline characteristics were described using medians and interquartile ranges (IQRs) for continuous variables and counts and percentages for categorical variables. Baseline characteristics were compared for patients starting TDF correctly and those started in error: continuous variables were assessed for skew and as all demonstrated non-normality they were compared using the Wilcoxon rank-sum test while proportions were compared using the Fisher's Exact test. The prevalence of baseline renal insufficiency (CrCl <50 ml/min) was determined and univariate and multivariate logistic regression models were built to calculate crude and adjusted odds ratios comparing CrCl <50 ml/min versus CrCl ≥50 ml/min. Multivariate models were built through backwards elimination to determine risk factors for reduced CrCl. Screening algorithms with different thresholds were developed using risk factors determined in the multivariate model. The number of patients needing screening and the number of cases with a CrCl <50 ml/min missed were calculated for each of these algorithms. Median change in CrCl from baseline for those initiated on TDF was calculated at 6 and 12 months. All reported P values are exact and two-tailed, and for each analysis a P-value <0.05 was considered significant.

The analysis was approved by the independent Ethics Review Board of Médecins Sans Frontières [9]. Individual patient consent was not sought because the analysis was based on routine clinical data, consistent with norms of the Ethics Review Board for secondary analyses. All patient information was entered into the database using coded identification numbers, and no information that could reveal patient identity was entered into the database.

## Results

### Program performance

566 patients with a baseline CrCl≥50 ml/min were started on TDF. Creatinine monitoring was performed in 57.1% of patients (298/522) still in care at six months, 47.3% of patients (192/406) still in care at 12 months, and 26.6% of patients (25/94) patients still in care at 18 months. A total of 2301 creatinine measurements were performed at baseline and during follow-up. CrCl was not documented for more than one third of measurements (824, 36%). The overall median difference between nurse- and formula-calculated CrCl was 1 ml/min (IQR 0–2). However, 268 nurse-calculated CrCls were outside ±10% of the computer-calculated CrCls. There was a significant improvement of nurse-calculated CrCls over time. 27.3% of nurse-calculated CrCls were outside ±10% range of the computer-calculated CrClS in the first half year, 20.6% in the second half year, 13.7% in the third half year and 9.7% in the last half year (p<0.01).

### Baseline characteristics

Baseline creatinine was measured for 933 adults prior to initiating ART. Median age was 40.6 years (IQR 32.6–50.4), the majority (62.5%) were women and median CD4 count was 209 cells/uL (IQR 119–282). 135 (14.5%) presented with normal renal function, 475 (50.9%) with mild renal impairment, 311 (33.3%) with moderate impairment, and 12 (1.3%) with severe impairment. Baseline characteristics of patients who were initiated on tenofivir (n = 566) are summarized in Table 1.

**Table 1:** Baseline characteristics of patients started on Tenofovir.

Variable	Patients started on TDF despite creatinine clearance <50 ml/min N = 15	Patients started on TDF with creatinine clearance >50 ml/min N = 566	p
Baseline creatinine clearance	45 (36–47)	73 (63–84)	–
Age (median, IQR)	50.7 (44.4–56.7)	38.9 (32.4–48.0)	0.0002
Men (N, %)	3 (20.0%)	271 (47.9%)	0.04
CD4 (median, IQR)	240 (209–297)	211 (120–282)	0.20
TB at ART initiation (N, %)	2 (13.3%)	102 (18.0%)	1.0
WHO stage 4 (N, %)	1 (6.7%)	44 (7.8%)	1.0
Opportunistic infection at ART initiation	0 (0%)	22 (3.9%)	1.0

### Risk factors associated with reduced baseline creatinine clearance

A total of 176 (18.9%) presented with a baseline CrCl <50 ml/min, the threshold at which TDF would be contraindicated. Univariate analysis showed that CrCl <50 ml/min was more likely in women, older patients, and patients with more severe immunosuppression (Table 2). Tuberculosis at the time of ART initiation and clinical stage IV were not associated with abnormal baseline renal function. Mutivariate analysis showed that patients aged ≥50 years were 11.4 times more likely to have a CrCl<50 ml/min compared to patients aged <30 years (95% confidence interval (CI) 5.44–23.98) Men were 0.36 less likely than women to have a CrCl <50 ml/min (95%CI 0.23–0.55). Having a CD4 count of <50 cells/uL increased the risk of renal insufficiency by 2.35 times compared to having a CD4 count >200 cells/uL (95%CI 1.33–4.16).

**Table 2:** Variables associated with creatinine clearance <50 ml/min (N = 933).

Variable		Creatinine Clearance >50 N = 757	Creatinine Clearance <50 N = 176	Unadjusted OR	Adjusted OR
Age (years)	≤30	147 (19.4)	10 (5.7)	1	1
	31–35	144 (19.0)	15 (8.5)	1.53 (0.67–3.52)	1.76 (0.73–4.24)
	36-40	119 (15.7)	10 (5.7)	1.24 (0.50–3.07)	1.73 (0.67–4.45)
	41-50	196 (25.9)	55 (31.3)	4.13 (2.03–8.36)	5.08 (2.40–10.74)
	>50	151 (20.0)	86 (48.9)	8.37 (4.19–16.74)	11.42 (5.44–23.98)
Gender	Women	444 (59.3)	134 (76.1)	1	1
	Men	308 (40.7)	42 (23.9)	0.46 (0.31–0.67)	0.36 (0.23–0.55)
TB at initiation	No	626 (82.7)	155 (88.1)	1	
	Yes	131 (17.3)	21 (11.9)	0.65 (0.40–1.01)	
WHO stage	1 or 2 or 3	697 (92.1)	163 (92.6)	1	
	4	60 (7.9)	13 (7.4)	0.92 (0.50–1.73)	
CD4*	>201	392 (52.1)	93 (55.0)	1	1
	200-101	198 (26.8)	35 (20.7)	0.75 (0.49–1.14)	0.74 (0.47–1.17)
	100-51	74 (10.0)	16 (9.5)	0.91 (0.51–1.64)	1.35 (0.72–2.53)
	≤50	74 (10.0)	25 (14.8)	1.42 (0.86–2.36)	2.35 (1.33–4.16)

*26 missing values

### Screening algorithms

Using these associations, we developed algorithms for baseline screening of creatinine. The aim of the algorithms, which are summarized in Table 3, were to demonstrate the numbers needing to be screened versus the proportion of cases with CrCl <50 ml/min that would be missed by their application. Scenarios 3 and 5 would both mean that 25% of the cohort would be excluded from screening but only 8% (14) of cases with baseline CrCl <50 ml/min would be undetected – 1.5% of the total cohort. Only 2 of the undetected cases had a CrCl <40 ml/min.

**Table 3:** Algorithms for baseline screening of creatinine.

	Scenario 1	Scenario 2	Scenario 3	Scenario 4	Scenario 5	Scenario 6
	No creatinine for individuals <30 and CD4>200	No creatinine for individuals <35 and CD4 >200	No creatinine for individuals <40 and CD4>200	No creatinine for individuals <30 and CD4>100	No creatinine for individuals <35 and CD4>100	No creatinine for individuals <40 and CD4>100
Individuals missed with Creatinine clearance<50 (%)	3 (1.8%)	10 (5.9%)	14 (8.3%)	4 (2.4%)	14 (8.3%)	19 (11.2%)
Number of individuals who would not need to be screened for Cr clearance (%)	87 (9.6%)	162 (17.8%)	227 (25.0%)	120 (13.2%)	231 (25.5%)	333 (36.8%)

### Renal function during follow-up and toxicity

Renal function improved during follow-up with a median change in CrCl of +5 ml/min (IQR 8–20) at 6 months (120–240 days) and +7 ml/min (IQR -3-22) at 12 months (240–480 days). 31 (5.5%) of the 566 patients initiated on TDF with a baseline CrCl≥50 ml/ml developed toxicity (CrCL <50 ml/min) during follow-up. The majority of these patients (28 of 31) had a drop in CrCl of less than <10 ml/min below the 50 ml/min threshold. Only 1 patient developed severe renal impairment (CrCl<30 ml/min). 29 of the 31 patients with renal toxicity during follow up remained in care, 1 patient was lost to follow-up and 1 died. The death was not related to TDF toxicity. A third of patients with renal impairment during follow-up (10 out of 29) were switched from a TDF-containing regimen to an AZT-containing regimen according to national guidelines. 19 remained on TDF despite renal impairment; among these, 17 had a CrCl of 40–50 ml/min and 2 had a CrCl of 30–40 ml/min at the time of renal impairment. Renal function improved (CrCl≥50 ml/min) in all but 3 of these patients who remained on TDF.

15 patients were started in error on TDF despite having a baseline CrCl <50 ml/min (range 30–49). When compared to patients who were started correctly on TDF, this group comprised more women, and older patients (p<0.05) (Table 1), consistent with the risk factors for low CrCl identified in multivariate analysis. In five cases the calculation was not performed, 6 were calculated incorrectly and the remaining 4 were started on TDF despite a correct CrCl calculation being performed. Among these patients, 3 were subsequently switched to AZT. Among those who remained on TDF, 6 experienced an improvement of renal function (CrCl≥50 ml/min), 3 had a stable renal function (CrCl 40–50 ml/min) and 3 had no further creatinine documented. None of these patients developed severe renal impairment (CrCl<30 ml/min).

## Discussion

In our study TDF was contraindicated in almost a fifth (18.9%) of patients due to a baseline CrCl of <50 ml/min. Implementing the protocol for CrCl follow-up both in terms of samples being drawn and correct calculation of CrCl in this setting was a challenge. Despite this, and the high median age of this cohort, documented toxicity was rare and mild.

Reported baseline renal function among HIV-infected patients in African settings is variable. Prevalence of mild renal insufficiency ranges from 24% in Zambia to 41.2% in Malawi, moderate insufficiency ranges from 7.6% in Zambia to 21.8% in Malawi, and severe insufficiency was under 2% in all studies [10]–[12]. Estimating the proportion of those who are not eligible for TDF according to currently accepted criteria of CrCl <50 ml/min is important for estimating the procurement needs for alternative regimens. For settings where TDF-initiation without baseline screening is proposed, consideration of the proportion with baseline renal impairment should be given to assess the degree of risk of potential toxicity.

In our study, older age and lower baseline CD4 count were associated with a CrCl <50 ml/min. (The relatively high baseline CD4 compared to other cohorts in southern Africa [13] is explained by the fact that Lesotho adopted guidelines for early initiation at CD4 <350 cells/uL in 2007.) These associations were used to develop algorithms that may reduce the numbers needing screening whilst limiting the risk of missing patients who may be at risk if given TDF. However, our study was limited by the limited number of baseline variables available for analysis; other studies have found that low hemoglobin (Hb<8) and body mass index (BMI<16) were associated with abnormal baseline renal function, in addition to older age and lower CD4 [10]. We plan to undertake additional studies including a broader range of covariates. Further multivariate analyses are needed to better define cost-effective and resource-appropriate algorithms to decide on the need for baseline renal monitoring to be carried out, including a broad range of potential risk factors such as diabetes, hypertension, and hepatitis co-infection. Such algorithms may aid policy makers to more clearly balance the cost of creatinine screening against the risks of missing cases more susceptible to toxicity.

In this community cohort, creatinine monitoring was not consistent and the accuracy of the calculation was poor. Implementation of TDF at primary care facilities in which lesser-trained health cadres will be responsible for routine care provision thus needs to be accompanied by either simplified visual tools for assessing the CrCl or by protocols to ensure that the calculation is performed electronically at the laboratory. In general, renal function improved on TDF and renal toxicity associated with TDF in our cohort was rare and mild. Only one case of incident severe renal impairment was reported, while among those patients who initiated TDF despite baseline renal impairment, there were no cases of further deterioration in renal function. A recent meta-analysis of 17 studies (10889 participants) found that TDF use was associated with a statistically significant loss of renal function compared to non-TDF containing regimens, but that the clinical magnitude of this effect was modest [14]. These findings are consistent with post marketing (industry sponsored) adverse-event surveillance which found that 0.2% of patients on TDF developed severe renal dysfunction. In Zambia, where TDF has been adopted as the preferred first-line since mid-2007, less than 3% of patients initiating TDF are reported to have had a CrCl decline to <50 ml/min [15]. Likewise, data from the DART trial [16], a prospective randomised clinical trial conducted in an African setting, showed a low (1.3%) rate of severe renal impairment through 96 weeks, with no difference in TDF-containing and non TDF-containing regimens.

The management of TDF toxicity in resource-limited settings, where care is often delivered by lesser-trained health staff at the primary care level, needs to be carefully defined to avoid both unnecessary switches or prolonged exposure to potential toxicity. In our cohort, the majority of those who developed a CrCl <50 ml/min dropped by less than 10 ml/min and subsequently returned above the threshold. Similarly, a proportion of those who dropped to 40–50 ml/min may have been unnecessarily switched if renal function had been rechecked after treating factors causing acute renal impairment such as diarrhoea, vomiting, or a urinary tract infection. Whil the use of TDF without dose reduction strategies in patients with a CrCl <50 ml/min needs further study, outcomes for those who were started on TDF in error with CrCl <50 ml/min in our programme are reassuring for programmes considering TDF-initiation without baseline renal assessment.

The strength of our study lies in the fact that we used data from a routine programme setting in an established programme, which provides a useful compliment to data derived from more controlled and better resourced research settings. However, the use of operational data comes at a cost of missing data and a limited number of covariates for analysis. Our study was further limited by the lack of consensus regarding which formula to use to calculate the CrCl (Cockcroft Gault vs MDRD) and compare with the gold standard [17]–[18]. Further research is needed to resolve this issue.

In summary, our study lends further evidence to previous reports from Africa that TDF-associated renal toxicity is rare and is in many cases a transient concern. Further studies to assess the safety of use of TDF at lower CrCl thresholds should be undertaken. The use of an evidence-based algorithm to rationalise the use of creatinine screening at baseline may serve as an important practical tool in countries choosing to switch to a TDF-based first line where resources for laboratory monitoring may be limited. Given the low levels of toxicity observed in this and other clinical settings, and considering the burden and cost of laboratory monitoring in under-resourced, high HIV-burden settings, the possibility of TDF implementation without any form of renal monitoring should not be ruled out.
